# On the Brink, a Population of Hedgehogs in Central London

**DOI:** 10.1002/ece3.72867

**Published:** 2026-01-07

**Authors:** John Gurnell, Nigel Reeve, Bryony Cross

**Affiliations:** ^1^ School of Biological and Behavioural Sciences Queen Mary University of London London UK; ^2^ 2 Paxton Gardens, Woking Surrey UK; ^3^ The Royal Parks, The Old Police House, Hyde Park London UK

**Keywords:** *Erinaceus europaeus*, hedgehogs, population surveys, urban park, volunteers

## Abstract

Numbers of the West European hedgehog ( *Erinaceus europaeus* ) have significantly declined in Britain and Europe over the past 50 years. In central London, they were once common in the Royal Parks but are now confined to The Regent's Park (166 ha), where they are isolated from other populations. Between 2014 and 2023, hedgehog numbers and spatial distribution were studied through twice‐yearly nocturnal searches in spring and autumn by volunteers using torches and thermal cameras, supported by ZSL Veterinary Services and the Garden Wildlife Health Project. Hedgehogs were tagged, examined and released. During the first 6 years, the population averaged 28 individuals (range: 13–38), with seasonal peaks in autumn. However, from 2020, numbers dropped, reaching just six in spring 2023 and slightly recovering to 10 by autumn. Breeding success was moderate to low and survival rates poor, raising concerns about long‐term viability. Of the 88 dead or fatally injured hedgehogs found, 59% showed signs of predation—mainly by foxes. Nearly all juvenile deaths (96%) were caused by predators. Other causes included road accidents, infections and drowning. Hedgehog distribution shifted over time. Initially widespread across the park, they began vanishing from the south and west in 2015, coincident with a population drop in 2016. Since then, they have mostly been found in the park's northeast quadrant. Factors likely to have contributed to the population's decline are considered. After a decade of monitoring, the remaining hedgehog population is critically small and faces extinction without urgent conservation action.

## Graphical abstract

A small breeding population of hedgehogs survives in central London's Regent's Park (166 ha). A 10‐year survey (2014–2023) revealed a decline from an average of 28 individuals to just six in spring 2023, driven by low breeding success and high mortality. Once widespread, hedgehogs are now confined to the park's northeast quadrant and face extinction without urgent conservation efforts.

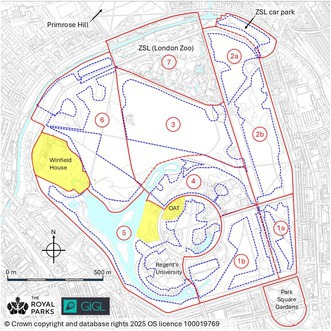

## 1 Introduction

The West European hedgehog ( *Erinaceus europaeus* ) is widely distributed across much of Europe but is experiencing a decline in numbers over most of its range. For this reason, it was listed as Near Threatened in The IUCN Red List of Threatened Species in 2023 (Gazzard and Rasmussen 2024). In Britain, one estimate of the decline in numbers nationally was from 1.5 million individuals in 1995 to 522,000 in 2016 (Mathews et al. 2018). Various factors are believed to have contributed to this decline including changes to agriculture that have resulted in habitat (e.g., hedgerows) and prey (e.g., invertebrates) loss (Hof and Bright 2009), predation, competition with badgers (Pettett et al. 2018; Williams et al. 2018) and roadkill (Huijser and Bergers 2000; Rondinini and Doncaster 2002; Wright et al. 2020). This decline is ongoing in rural areas, but there is recent evidence that numbers may have levelled off and are perhaps recovering slightly in urban areas (Wembridge et al. 2022), which may be related to a mix of gardens, recreational and other green spaces in urban areas providing a reliable source of food and cover, although road casualties are still an issue.

Despite being a relatively common mammal, it can easily go unnoticed, and its decline can be surprising in the absence of long‐term monitoring programmes for the species. This paper describes changes in numbers of hedgehogs in a small population in a Royal Park in central London between 2014 and 2023. It is one of only three hedgehog population monitoring studies of 7 years or more (Kristiansson 1990; Yu et al. 2025). In the early 1970s, hedgehogs were documented as being present in all five of London's central Royal Parks, Kensington Gardens, Hyde Park, Green Park, St. James' Park and Regent's Park (Simms 1974). However, they have since vanished from all except The Regent's Park (henceforth called the Park). The reasons behind the decline in hedgehog populations in central London remain unclear, but a high degree of habitat fragmentation and isolation and a deterioration of habitat quality are important factors to consider. Although the presence of hedgehogs in the Park was known at the start of the study, there was little understanding as to how many there were and how likely they were to persist since they constitute an isolated population (Gurnell et al. 2015; Turner 2024; Turner et al. 2022). In 2014, a study was initiated to gain an understanding of the ecology of hedgehogs in the Park by engaging volunteers, the local community and other stakeholders. Several internal reports on the survey's progress have been produced, primarily to provide feedback to the volunteer surveyors and the local community (see Gurnell et al. 2015, 2017, 2024). This paper presents findings from the 10‐year survey and examines whether they offer insights into the vulnerability of the hedgehog population to extinction.

## 2 Methods

### 2.1 The Regent's Park

**FIGURE 1:**
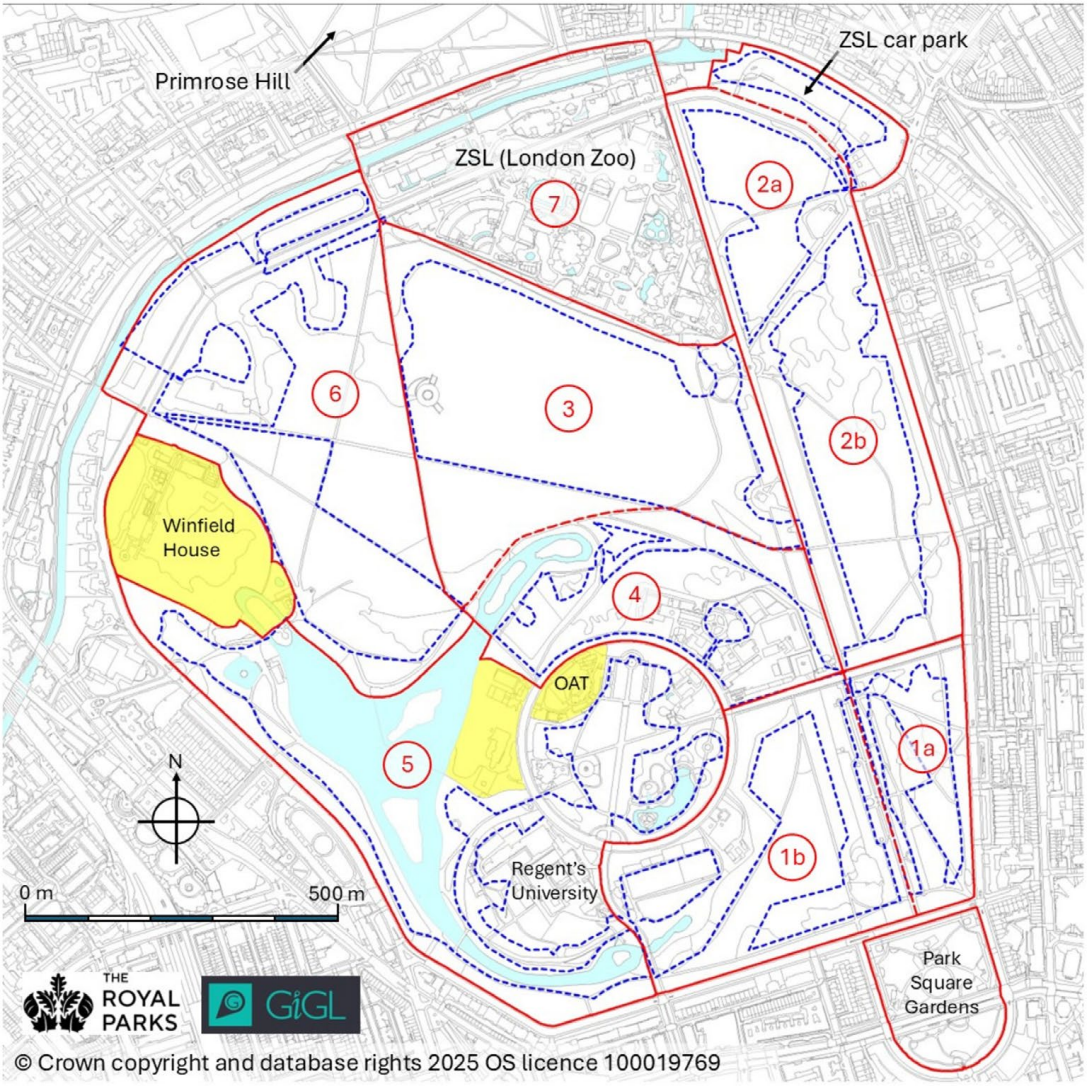
The Regent's Park (166 ha) showing (in red) the boundaries of seven survey zones (1–7). Spotlighting survey routes in each zone are the dashed blue lines. Zones 1 and 2 were divided into two sections (a, b) for carrying out the surveys. The areas shaded yellow were private and inaccessible or only occasionally accessible throughout the study. Permission for surveys to be carried out in the grounds of Regent's University was granted in 2015. OAT = Open Air Theatre.

The Park is located in north‐west Inner London and covers approximately 166 ha. It comprises a mix of formal and informal habitats, including sports pitches, extensive grasslands, woodland, scrub, hedgerows, wetlands and reedbeds, shrubberies, flower beds, a large rose garden, and a boating lake (about 6 ha). London Zoo (15 ha) has occupied the northern section of the Park since the first half of the 19th century. A separate but associated area, Primrose Hill (25 ha), lies across a main road to the north of the main park (Figure 1). A questionnaire survey of residents reported no sightings of hedgehogs in Primrose Hill since 2000 (Gurnell et al. 2015). The area was surveyed in 2015, but no hedgehogs were found, and it is therefore not included in the findings reported here. Four areas within the Park were privately owned and were not included in the surveys (Figure 1). Permission to search for hedgehogs in the grounds of Regent's University was granted in 2015. The nearest known hedgehog population to the Park is on Hampstead Heath, located over two kilometres to the north as the crow flies.

### 2.2 Fieldwork

Surveys for hedgehogs within the Park were carried out by volunteers each spring in May and each autumn in September between 2014 and 2023; the work was carried out under licence from Natural England (licence number 2014/SCI/0402 and subsequent‐related licences). Volunteers were recruited by The Royal Parks. Each volunteer received a comprehensive survey guide and risk assessment. Before each survey, they were trained to conduct spotlighting surveys, identify habitat types and handle captured hedgehogs safely. To standardise survey procedures, the Park was divided into seven zones. Owing to the high initial abundance of hedgehogs in Zones 1 and 2, each was subdivided into parts a and b to allow two teams to survey these areas simultaneously (Figure 1). Teams, comprising four to five volunteers led by a Project Team supervisor or a trained volunteer supervisor, were assigned to each zone and walked designated routes around their allocated zone in two shifts (early shift 21.00–24.00, late shift 00.30–04.00) on two consecutive Fridays in May (spring) and September (autumn). Members of the team were equipped with a powerful torch (LED Lenser P7.2 or similar) to search for hedgehogs and the remaining team member used a FLIR E60 TIC (Thermal Imaging Camera; available at https://www.flir.co.uk) (see Bowen et al. 2020). Additionally, all volunteers carried personal torches for safety reasons. Searching was carried out in all weather conditions. The grounds to the Zoo (Zone 7) were surveyed by Zoo personnel. Volunteers with torches were instructed to move slowly and quietly, scanning their surroundings and intermittently pausing to listen for sounds of rustling in the undergrowth or hedgehog activities such as courtship or fighting. The volunteer with the TIC would halt every 20–30 m to scan the environment using the manual focus on the camera. As part of a separate study, unmarked hedgehogs encountered during the radiotracking of selected individuals between the two Fridays of each survey period in 2014 and 2015 were marked (see Gurnell et al. 2015).

The locations of captured hedgehogs were recorded using a handheld GPS and marked on a map. The habitat at each capture site was classified into one of 11 categories that reflected park management and could be easily identified by the surveyors after training: bare artificial habitat, bare soil, short grass (< 10 cm), tall grass (> 10 cm), tussocky rough grassland (> 10 cm), hedgerow, planted shrubbery, scrub, sports pitches, tall herbs and woodland floor. The hedgehogs were sexed, weighed (if an animal was weighed more than once within a survey, the mean weight was used), their body circumference measured (around the head‐tail midline when the hedgehog was in a rolled‐up position), examined for injuries and ectoparasites, marked and released. In 2014, hedgehogs were uniquely marked with colour combinations of 5–10 mm lengths of coloured plastic (polyolefin) electrical sleeving glued over five individual spines in each of one or more areas of the dorsal pelage. In 2015 this system was replaced with six yellow plastic sleeves (1.6 mm internal diameter polyolefin sleeving) bearing identical pre‐printed numbers glued to the spines on the nape (for details, see Reeve et al. 2019). Lost sleeves on recaptured hedgehogs were replaced with additional sleeves bearing the same number so that the hedgehog always had six markers when released.

Covid restrictions meant that the Park could not be surveyed in spring 2020, and restrictions in the autumn of that year on the number of people who could be in the Park at any one time meant that the surveys were slightly modified. Only two zones could each be searched by up to three people per zone (including supervisor) on any one night. The surveys, therefore, took place over three nights to cover all six zones within the Park. Numbers of individuals captured during this survey period should be treated with caution. During the first survey in spring 2014, the Zoo Car Park (ZCP) in Zone 2 was not searched. A large number of individuals were found in the ZCP in the autumn of that year, and therefore numbers of individuals captured in spring 2014 are likely to be lower than they should have been. In 2014 and 2015, selected individuals were marked with radio/GPS tags and were tracked by volunteers at night between the two survey Fridays (Gurnell et al. 2015). Any unmarked hedgehogs found during these tracking studies were marked and released and these animals have been included in the totals of individuals captured during these surveys.

The Zoological Society of London (ZSL) Veterinary Services team was on standby during the surveys and all injured hedgehogs were taken to ZSL's veterinary centre within the Park for examination. If a hedgehog could be treated, it was released back into the Park, usually within 2–3 days. If the hedgehog was too badly injured, it was euthanised. Two hedgehogs were not deemed well enough to release into the Park but were sufficiently well to be placed with an experienced hedgehog carer. Injured or dead hedgehogs found within the Park during the surveys, or at other times by members of the public or park/zoo staff, were logged. Dead or euthanised hedgehogs were subject to a postmortem examination to try and ascertain the cause of injury or death by members of the Garden Wildlife Health Project based at ZSL's Institute of Zoology (https://www.gardenwildlifehealth.org/).

### 2.3 Data Analysis

We estimated the number of hedgehogs present during each survey in Excel using Chapman's modification of the Lincoln–Petersen estimator for small samples (Southwood and Henderson 2000), based on Capture–Mark–Recapture (CMR) data from the two sampling nights in each survey. To explore survival and recruitment of individuals, we have used the Minimum Number known to be Alive (MNA) at the time of each survey which includes individuals that were captured before and after the survey but not at the time of the survey. During the autumn surveys, it was not possible to determine the age of individuals in the field by their appearance, so unmarked hedgehogs < 700 g when first captured were recorded as juveniles. Although the weight of 700 g was somewhat arbitrary it was based on experience; adults should be > 700 g in early September (having recovered from any weight loss during the previous hibernation period), and any young born from late May onwards are unlikely to achieve 700 g by early September. Only three of 141 (2%) marked adults weighing under 700 g were captured during the autumn surveys (660, 675, and 690 g), reinforcing 700 g as a practical cutoff between juveniles and adults. Juveniles caught in early September were likely born between May and the first half of July, excluding individuals born in the latter half of July or later. As a result, our autumn count of juveniles underrepresents actual recruitment. Many of the young born later in the season would appear in the following spring surveys when, if sufficiently well‐grown, they could not be distinguished from previously unmarked adults that were missed in the autumn.

The GPS locations of captured hedgehogs within the Park were taken with a Garmin handheld GPS tracker, marked on a map and each hedgehog was allocated to a survey zone. For any individual captured more than once during a survey, the arithmetic centre of the capture coordinates (the range centre) was used, even though a few individuals moved between zones during a survey. The term survival has been used throughout the paper, even though it was not known whether a marked individual that was not captured again had died or simply avoided capture. To understand the probability of hedgehogs dying at a particular age, survivorship curves based on the number of individuals alive at age *x* (denoted lx) and the mean life expectancy at age *x* (denoted ex) were calculated in Excel for juveniles first captured during the autumn pooled across 2014–2021 using standard life table analysis (e.g., Krebs 1999).

For each survey, yearly range centres were classified by zone, and the data were examined using Ranges 9 (Anatrack Ltd.) to determine how far the range centres of recaptured individuals had shifted between years. This allowed us to assess whether shifts in distribution between years could be explained by animals moving between zones. The median distance moved by recaptured animals was calculated across all years. Individuals whose interyear movement distances fell within the top 25% (above the upper quartile) were classified as long movers.

Generalised Linear Models were used to explore body weights according to sex and season. Nonparametric Mann–Whitney tests were used to analyse percent population losses between 2014 and 2019. Statistical analyses were carried out in XLSTAT and Minitab.

## 3 Results

### 3.1 Numbers of Individuals

**FIGURE 2:**
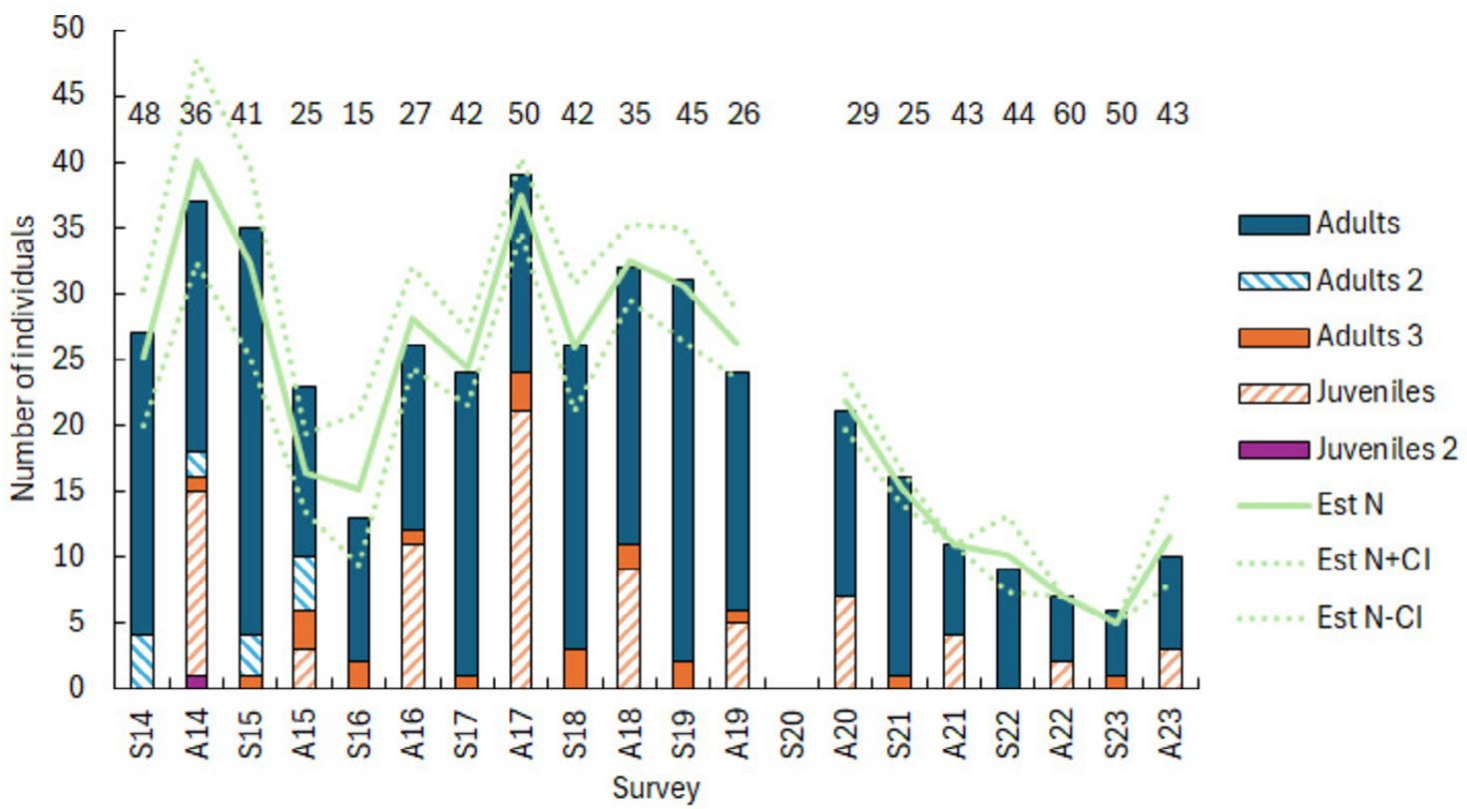
Bars—minimum number of hedgehogs known to be alive (MNA) at the time of each survey in the Spring (S) and Autumn (A) each year from 2014 to 2023. No survey was carried out in Spring 2020 because of Covid restrictions. Juveniles 2 and Adults 2 refer to the numbers of hedgehogs captured on non‐survey nights during the radiotracking studies carried out in 2014 and 2015. Adults 3 refer to numbers of hedgehogs captured before and after the survey but not captured during the survey itself. Fourteen individuals were in the ZCP in Autumn 2014, and the number captured in Spring 2014 is likely to underestimate the numbers of hedgehogs in the Park at that time. Dashed line = CMR population estimates, dotted lines = CMR ± 95% confidence intervals. The numbers above each bar refer to the percentage of adults that were male in each survey.

During 2014 and 2015, between two and four individuals were captured during radiotracking studies carried out during the week between the two successive survey Fridays in each autumn and spring survey, but were not captured during the surveys themselves (Figure 2). Generally, the numbers of marked individuals not captured during a survey, but captured before or after, were low and mostly female (mean no of females not captured = 1.18, SD = 1.01, *N* = 17; male = 0.12, SD = 0.33, *N* = 17; Figure 2).

Across the study, the mean CMR estimate was almost identical to the mean MNA estimate (mean CMR = 21.9 vs. mean MNA = 21.9; paired *t*_18_ = 0.095, *p* = 0.926), and the observed trends were similar (Figure 1). Across the study, the MNA can be viewed in two periods. Between 2014 and 2019, the hedgehog population (adults and juveniles) fluctuated around an average of 28 individuals (SD = 7.2, *N* = 12) with peaks in the autumns of 2014 and 2017 (Figure 2). Between these peaks, the population dropped to a low of 13 individuals in spring 2016, comprising 2 males and 11 females. Since the autumn of 2020, the population declined to a second low of just six individuals, three males and three females, in spring 2023. The population recovered slightly from this low to 10 individuals at the end of the study in autumn 2023.

Except for spring 2014 and autumn 2017, the numbers of adult males were consistently lower than the numbers of adult females captured over the first 6 years of the study and dropped to just two males in spring 2016 (Figure 2). Numbers of adult males captured were generally lower than females towards the end of the study, although the population was very small.

### 3.2 Recruitment and Survival

**FIGURE 3:**
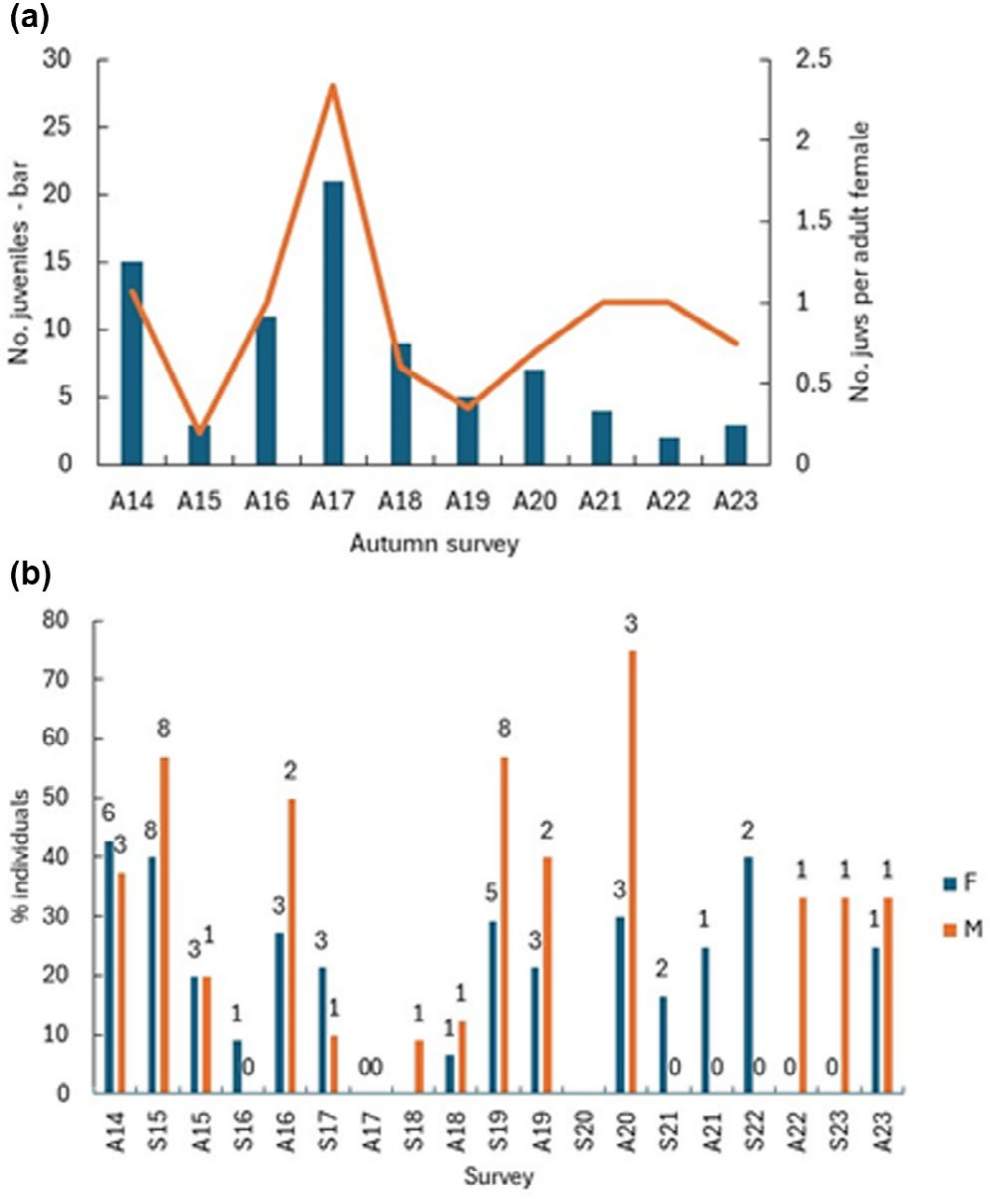
(a) The number of juveniles (< 700 g) captured (bars), and the number of juveniles captured per adult female each autumn (line) from 2014 to 2023. (b) The per cent of adults that were unmarked in each survey; the figures above the bars are the number of individuals.

Based on juveniles captured in early September, breeding occurred in every survey year. Across the study, the number of juveniles per adult female averaged 0.90 (SD = 0.582, *N* = 10), with a low of 0.2 in autumn 2015 and a high of 2.3 in autumn 2017 (Figure 3a). At other times this value ranged from 0.4 to 1.07 juveniles per adult female with no clear pattern and no link to the number of adult females captured. Year 2017 showed the strongest recruitment and, together with recruitment in 2016, helped the population recover from the low capture numbers recorded in spring 2016.

Unmarked adult hedgehogs were recorded in most surveys (Figure 3b). In autumn 2014, five of the nine unmarked adults were captured in the Zoo Car Park, a site not surveyed the previous spring. Excluding that year, the average number of unmarked adults (male and female) captured in autumn was 2.9 (SD = 2.15, *N* = 9). The spring average was higher at 5.0 individuals (SD = 6.00, *N* = 8), with clear peaks in spring 2015 and spring 2019, likely reflecting strong late‐season recruitment in 2014 and 2018. When these 2 years are excluded, the spring average falls to 1.8 (SD = 1.17, *N* = 6), indicating that late recruitment was generally low in many years.

**FIGURE 4:**
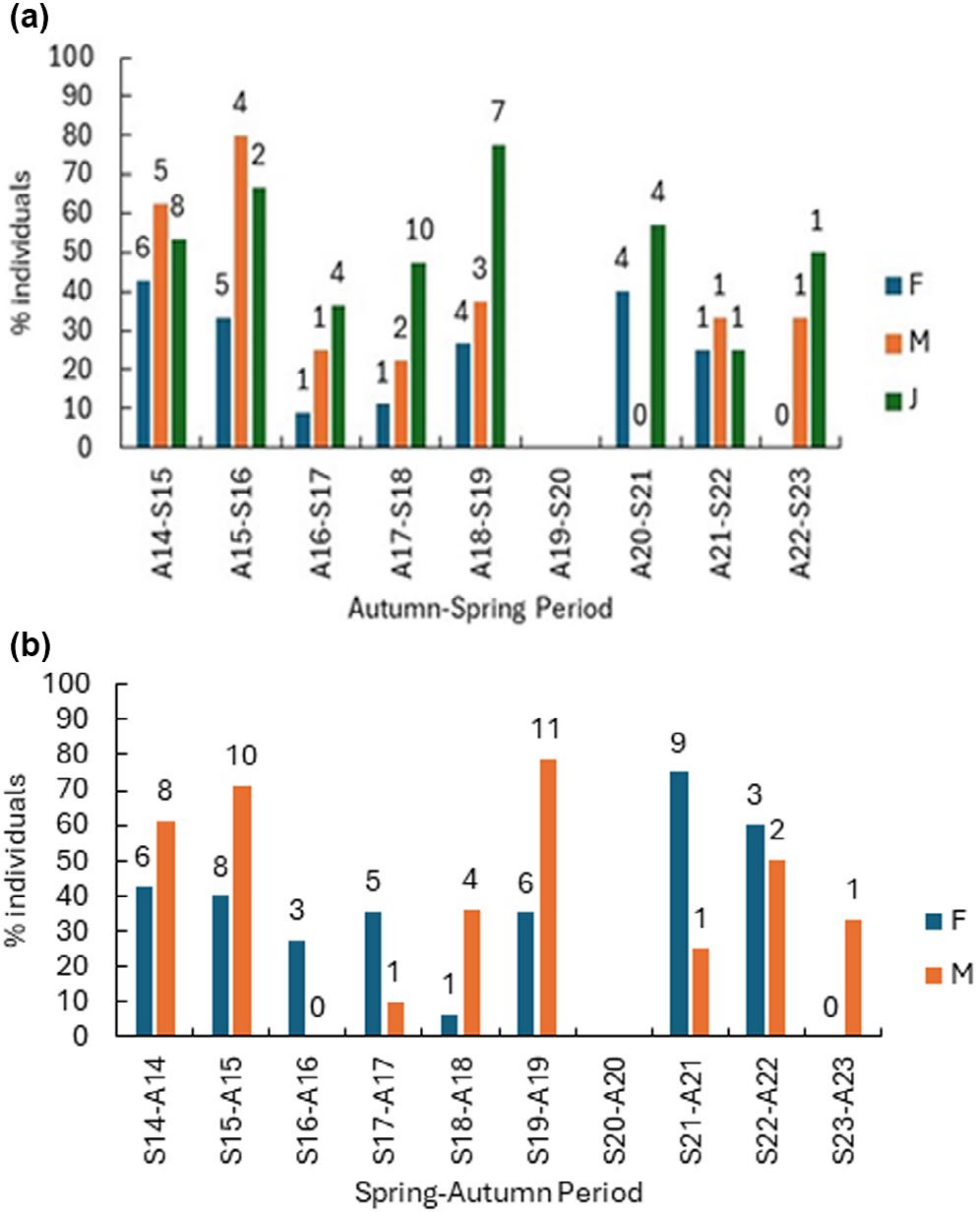
(a) Per cent of individuals lost overwinter (Autumn‐Spring period) losses of numbers of individuals and (b) per cent of individuals lost over‐summer (Spring‐Autumn period). The figures above the bars are the numbers of individuals. MA = male adults, FA = female adults, J = juveniles. No survey was carried out in Spring 2020 because of Covid restrictions.

The number of marked animals not captured again (i.e., were lost from the surveyed population) varied considerably from season to season and year to year (Figure 4). For a more detailed analysis, we focused on the period between 2014 and 2019 before the decline at the end of the study when sample sizes were small. The number of adult male and female overwinter losses was similar, although the percentage of males lost was higher than the percentage of females lost; this difference was not significant (Table 1; Mann–Whitney *U* = 7, *p* = 0.310). Average juvenile losses overwinter were the same as adults (sexes combined), but their percentage was higher though not significant (Table 1; *U* = 3, *p* = 0.056). Over‐summer losses were slightly higher in adult males than females, though per cent losses were not significantly different (Table 1; per cent losses *U* = 13, *p* = 0.485). The number of adults (sexes combined) and per cent of adults lost were slightly higher over‐summer than overwinter (Table 1; per cent losses *U* = 21, *p* = 0.329).

**TABLE 1:** Mean numbers and percentage numbers of adult male (AM), adult female (AF) and juvenile (J) hedgehogs lost overwinter (autumn to the following spring) and over‐summer (spring to the following autumn), 2014–2019. Tot = total, Ad =adults.

		No. AF	% AF	No. AM	%AM	Tot Ad	% Ad	No. J	% J
Overwinter losses	Mean	3.4	24.6	3.0	45.4	6.4	31.1	6.2	56.4
*N* = 5 years	SD	2.30	14.46	1.58	25.03	3.85	16.39	3.19	16.20
Over‐summer losses	Mean	4.8	31.3	5.7	43.0	10.5	37.8		
*N* = 6 years	SD	2.30	14.46	1.58	25.03	6.60	16.98		

Between 2020 and 2023, population sizes were low and numbers lost were low (Figure 4), although it is notable that percentage losses were often quite high (average adults M + F overwinter 26%, juveniles 44% and adults M + F over‐summer 44%).

**FIGURE 5:**
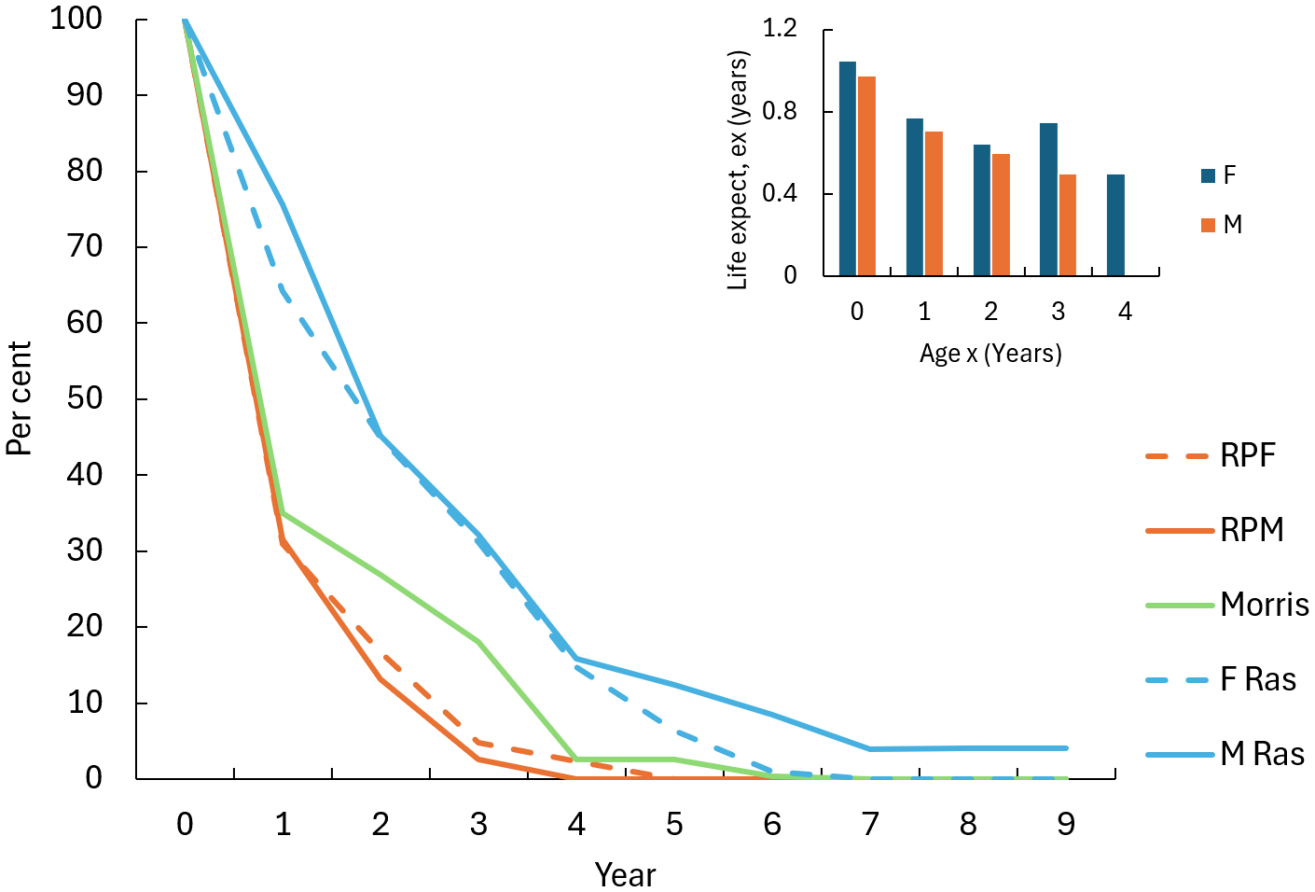
Survival of cohorts of male and female juveniles captured each autumn between 2014 and 2021 combined data. RPM = Male (*N* = 38), RPF = Female (*N* = 42). Ras = Rasmussen et al. (2023); *N* F = 109, *N* M = 177, Morris = Morris (1969, 2018, *N* = 244). Insert—expectation of future life (years), ex, for hedgehog of age *x* (years). M = male, F = female.

In the Park, the survival of autumn captured juveniles was low; one female hedgehog survived to Age 4, and only two females and one male reached Age 3 (Figure 5). Survival rates were much lower than those reported by Rasmussen et al. (2023), and the Park hedgehogs also showed poorer survival than the estimated rates for individuals aged 2–4 years in Morris (1969) study (Figure 5). On average, the expectation of future life (life table statistic ex) when a juvenile first enters the population (notionally age 0 years) was about 1 year in both males and females and it declined thereafter (Figure 5).

### 3.3 Adult Body Weight

The mean weight of all adult hedgehogs captured during the 10‐year study was 892 g (*N* = 316, SD = 164.2 g). There was considerable variation in body weight from survey to survey without any clear pattern, and generally body weights from 2020 (when numbers declined) did not appear any different to those at any other time of the study. However, pooling the data for males and females for spring and autumn revealed that hedgehogs were heavier in autumn than spring (mean weight spring = 843 g, *N* = 175, SD = 158.2 g, mean weight autumn = 952 g, *N* = 141, SD = 150.2 g; F = 36.64, *p* < 0.001). Females tended to be slightly heavier than males, but this was not significant (mean weight females = 902 g, *N* = 190, SD = 177.0 g, mean weight males = 877 g, *N* = 126, SD = 142.2 g; F = 1.06, *p* = 0.305), and there was no interaction between season and sex (F = 0.17, *p* = 0.677).

### 3.4 Deaths

**FIGURE 6:**
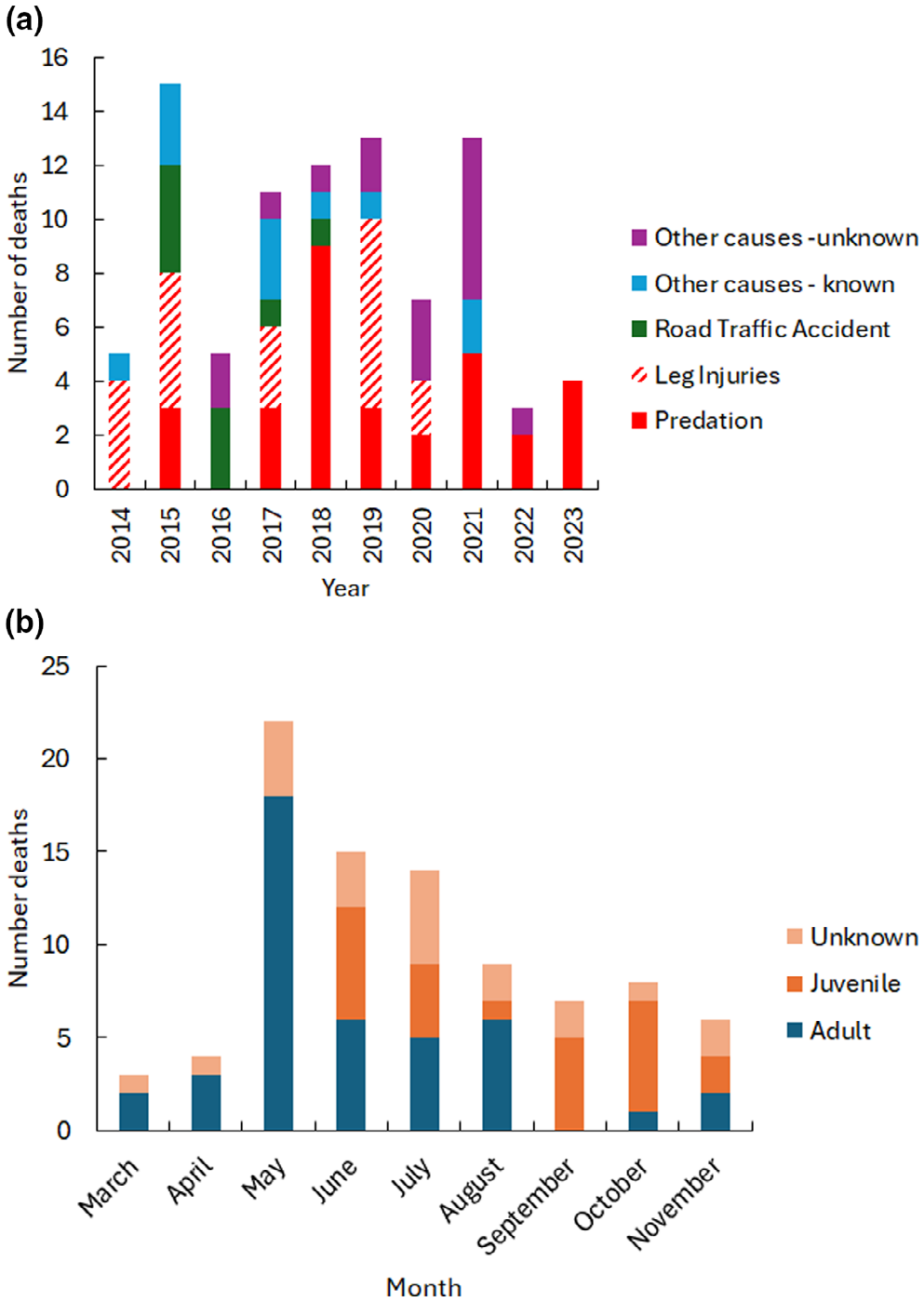
(a) Hedgehog deaths each year, (b) Adult and juvenile deaths according to month.

Over the course of the 10‐year study, six hedgehogs were found that required veterinary care. Two of these were later placed in homes with caregivers and the other four released back into the Park. A further 78 animals were either discovered dead or had to be euthanised due to severe injuries, such as significant leg wounds. For some of the deaths, details such as the animal's sex, age, markings, the precise location of discovery or cause of death remain unknown. However, deaths could usually be attributed to factors such as road accidents, predation, leg injuries, disease, or drowning (Figure 6a). It is believed that most leg injuries were caused by predator bites, most likely from foxes or possibly dogs. Fifty‐three per cent of dead animals were tagged, representing a third of all animals marked during the study; 92% of the marked dead hedgehogs were adult.

During the study, most hedgehog deaths occurred in May, and all were adults when the age could be determined (Figure 6b) Later in the year, many deaths involved juveniles. Predation and predator‐related leg injuries were responsible for 96% of juvenile deaths, compared to 46% in adults. Additionally, over 80% of juvenile fatalities occurred in Zone 7, the Zoo grounds.

### 3.5 Location of Individuals Captured

**FIGURE 7:**
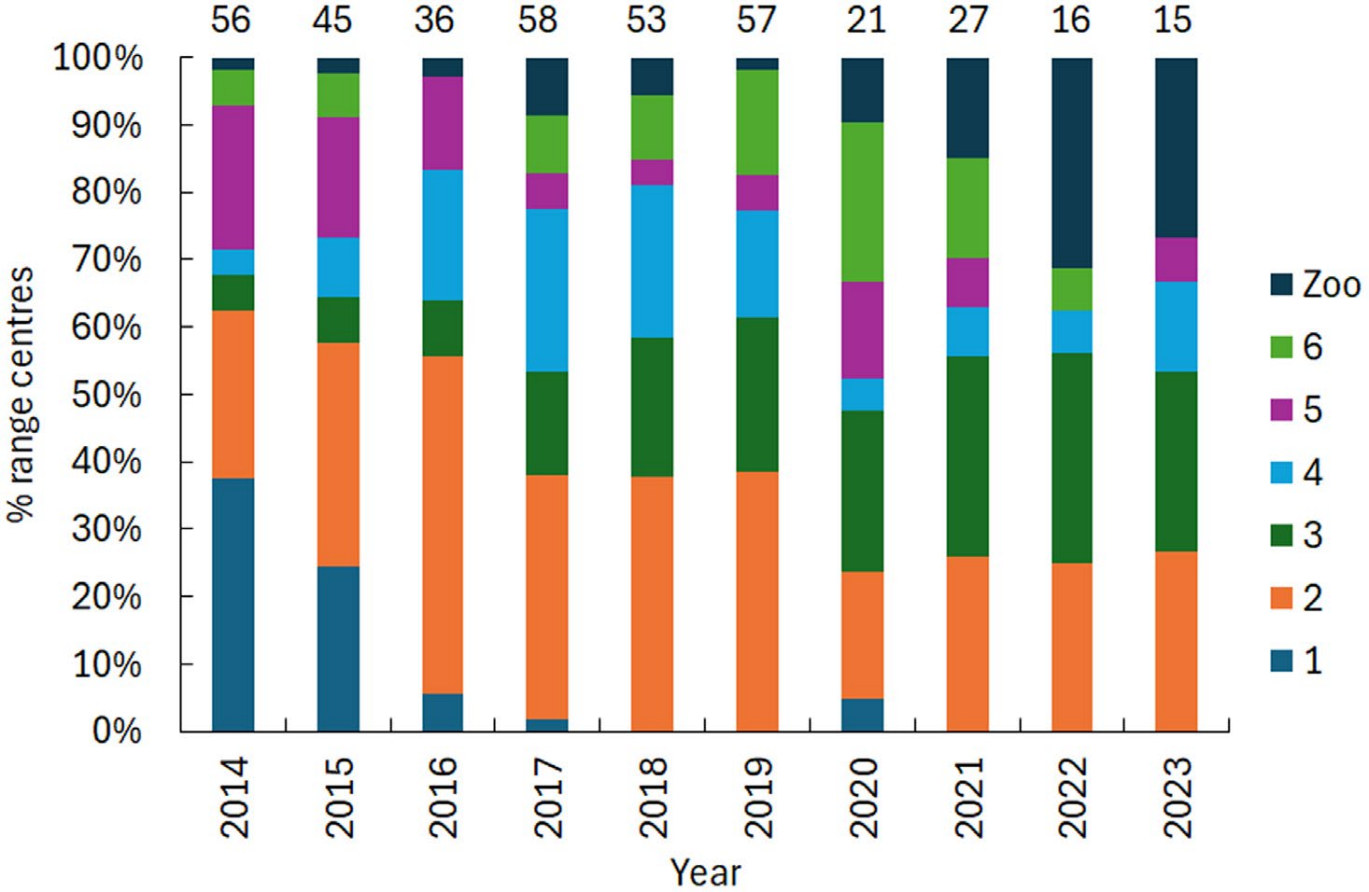
The percent range centres of hedgehogs in each zone in each year of the study. Numbers above the bars are the number of hedgehogs.

To illustrate the distribution of hedgehogs captured within the Park, spring and summer survey data were combined for each year. Since the study began in 2014, there has been a notable shift in the distribution of hedgehogs within the Park (Figure 7). In 2014 and spring 2015, Zones 1, 2, and 5 were identified as ‘hotspots’ with the highest proportions of hedgehogs. However, over 2015 there was a sharp decline in hedgehogs captured in Zone 1, since when it has never recovered. Numbers fell slightly in 2015 in Zone 5 and more steeply in 2016. In contrast, hedgehogs captured in Zone 2 increased and peaked in 2016 with a decline until 2020 and a slight increase thereafter. Within Zone 2, the ZCP became a ‘local hotspot’ accounting for 25% of all captures in autumn 2014 and 20% in spring 2015. However, hedgehog numbers declined over the following 5 years, and no hedgehogs were found in the ZCP from 2020 until the study concluded in 2023.

In other areas of the Park, captures in Zone 3 have increased over the study period, particularly around its periphery. Individuals captured in Zone 4 have fluctuated, while captures in Zone 6 peaked in 2016 but have been low at other times (Figure 7). Hedgehogs captured in the zoo grounds (Zone 7) were low until 2019 but have increased substantially towards the end of the study, indicating the growing significance of this area.

Over the 10‐year study, 41% of hedgehogs were recaptured the following year, while 59% were not recaptured and disappeared in situ (Table A1). The median distance moved between years was 142 m (*N* = 121). ‘Long movers’, individuals in the top 25% of inter‐year movement distances, are listed in Table A1. In this group of 30 hedgehogs (13 females, 17 males), two‐thirds (66%) moved less than 500 m. There was no consistent movement pattern across years or zones. Notably, the decline in numbers from 2015 to 2016, and the disappearance of animals from Zones 1 and 5, cannot be explained by movements away from these areas.

### 3.6 Habitat Check Area of Grassland Habitats

**FIGURE 8:**
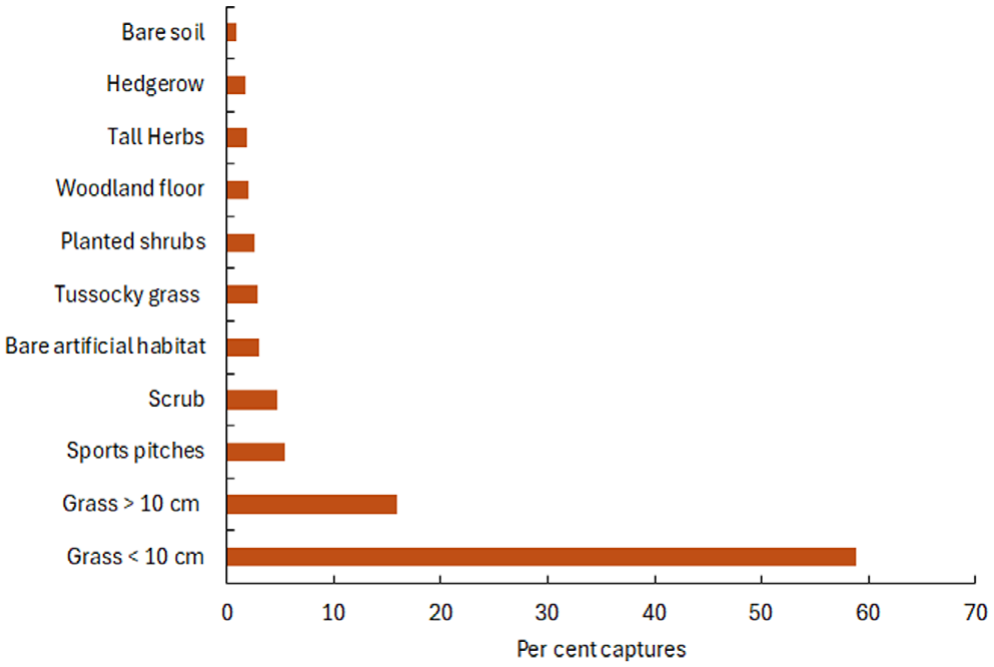
Percent of captures in each habitat pooled across 2014–2023.

Since the study began, 59% of all hedgehog captures have occurred in short grass less than 10 cm high (Figure 8). Sixteen percent of captures took place in grass taller than 10 cm, while 5% occurred on sports pitches and another 5% in scrub, with relatively few captures in other habitat types.

## 4 Discussion

Chapman's CMR estimates of population size closely matched the MNA results in each survey, indicating that the key assumptions of the CMR model (closed population, equal catchability, mark retention, no behavioural effects and adequate mixing; Southwood and Henderson 2000) were largely met. The MNA analysis showed that few individuals were missed, mostly females; possibly because, in Spring and Summer, females are generally less active and therefore encountered less often than males (Reeve 1994). However, the repeated capture of a small number of previously unmarked adults suggests the true population size was slightly higher than either estimate. Overall, we are confident that the survey methods effectively tracked hedgehog numbers within the Park over the 10‐year period, providing valuable insights into population trends and distribution changes.

The twice‐yearly survey schedule was disrupted by COVID‐19 restrictions, preventing the spring 2020 survey and leading to minor methodological changes in autumn 2020. Regent's Park remained open, but cafes and other venues were closed. There is no evidence that these restrictions directly affected hedgehogs, though a decline in numbers and shifts in distribution were observed after 2020.

Most active hedgehogs were recorded in open habitats such as short grassland, likely reflecting both easier detection and the preponderance of this habitat type within the Park. The areas of the different habitat types surveyed varied each year with park management, especially the extent of tall grass, tall herbs, planted shrubs and bare soil. However, short and tall grass areas combined with sports pitches covered about 90 ha (Regent's Park Grassland Management, Claudia Watts, personal communciation). Radiotracking studies from 2014 and 2015 (Gurnell et al. 2015) confirmed a strong preference for grassland, hedgerows, and rough ground. During the day, hedgehogs mainly used scrub, planted shrubberies and hedgerows for nesting. Huijser (2000) found that hedgehogs spent 53% of their active time in grasslands, over half within 5 m of a hedgerow or forest edge. Similarly, Parrott et al. (2014) reported higher densities on amenity grassland (0.47 ± 0.09 ha^−1^) than on pasture (0.04 ± 0.02 ha^−1^). In New Zealand, hedgehogs occurred across all urban habitat types but were less likely to be detected in forest fragments than in amenity parks (mostly lawn) and showed no preference for residential gardens (Miller et al. 2022). Other studies have linked habitat use to vegetation structure, habitat complexity and prey availability (Riber 2006; Haigh 2011; Haigh et al. 2013). Although our study did not measure prey abundance, the observed preference for grassland likely reflects greater food availability and ease of movement between foraging areas and potential mates.

### 4.1 Changes in Numbers and Distribution of Hedgehogs

Two phases were evident during the study. Between 2014 and 2019, numbers fluctuated but remained relatively stable, averaging 28 individuals. From autumn 2019 onward, numbers declined steadily until spring 2023. Within the first phase, the lowest count occurred in spring 2016 (13 individuals, 11 of them females), but this group recovered quickly, suggesting good recruitment potential. No comparable recovery followed the decline from 2020 to 2023.

During the first phase, hedgehogs disappeared from the south (zone 1) and declined in the west (zone 5) starting in summer 2015, contributing to the 2016 low. There was no evidence of dispersal from these zones to the north or east. The southern and western areas never recovered; thereafter, most records came from the eastern, central and northern parts of the Park. The Zoo Car Park was an early hotspot, though the reasons for its initial popularity remain unclear. Since 2014, the ZCP habitat has undergone significant disruption due to major projects, including the replacement of water mains and the construction of a Lorry Holding Park for the HS2 project near Euston Station. Numbers there began to drop in 2018, and none were captured in the final 4 years. This is possibly due to habitat changes or increased disturbance, although numbers elsewhere in the Park also declined.

By the end of the study, the Zoo grounds (Zone 7) became increasingly important, with higher capture rates and signs of breeding, including dead juveniles indicating local litters. Hedgehog‐friendly fence holes added in 2014–2015 likely supported movement and reduced disturbance, though fox predation likely caused many juvenile deaths. The Zoo area therefore appears to have served as a key breeding site.

### 4.2 Recruitment and Survival

Reproduction occurred every year, with population numbers typically higher in autumn than spring, except in later years when numbers fell sharply. The autumn surveys were held two to 3 months before hibernation and so are not influenced by reduced activity (cf. Miller et al. 2022). However, some late‐season juveniles may have been missed due to the early timing of autumn surveys, as unmarked individuals appeared the following spring. Thus the recruitment figures must be treated with caution. Recruitment varied throughout the study: it was moderate in 2014, 2016, 2017 and 2018 but insufficient to sustain long‐term growth. The highest recruitment, in 2017, was 2.3 juveniles per adult female; in other years it ranged from 0.2 to 1.0. There are few data on recruitment rates in the literature. Moore et al. (2023) reported similarly low reproductive rates across four study areas, with 0.43, 0.50 and 0.52 juveniles per female at three sites and none recorded at the fourth. In contrast, Kristiansson (1990) found a range of 1.33–4.13 juveniles per female (average 2.8) in Sweden.

Survival in the Park was lower than in most other studies. The average juvenile lifespan was just over 1 year, compared to 2.6 years for Danish males and 2.1 years for females (Rasmussen et al. 2023). Few individuals survived beyond age three, and only one reached age four. Our estimates, based on individually tracked hedgehogs, differ from age‐structure data from dead specimens (Morris 1969; Haigh, Kelly, et al. 2014; Parkes 1975). Haigh, Kelly, et al. (2014) reported a mean age at death of 2.10 years for females and 1.87 years for males, with an overall mean of 1.94 years. Most (87%) were aged 0–3 years; the oldest females were 9 years old (*N* = 2) and the oldest males were 8 years old (*N* = 2). In New Zealand, Parkes (1975) estimated an average lifespan of 1.97 years for adult hedgehogs (presumed to be 1 year old at the start of the study), based on surveys of populations in a mix of dairy pastures and pine plantations. Parkes also estimated that 20% of hedgehogs died over winter, whereas our observed overwinter losses were higher: 31% in adults and 59% in juveniles. Kristiansson (1990) found annual mortality of 47% in adults and 34% in juveniles, mainly during winter. In contrast, our data show slightly higher adult mortality in summer (38%) than in winter (31%), suggesting overwintering was not especially hazardous. Adult females were slightly heavier than males, and autumn individuals heavier than those in spring, but body weight did not correlate with population trends.

Camera‐trap surveys throughout the Park indicate high fox density (Carbone, personal communication) and predation was the leading cause of known deaths, responsible for 44% of adult and 92% of juvenile fatalities. These are likely due to foxes ( *Vulpes vulpes* ), although dog ( *Canis familiaris* ) attacks cannot be ruled out. Foxes probably preyed directly on juveniles but may have destroyed nests with young, although evidence is lacking. Casual camera trapping within the Park also recorded foxes and adult hedgehogs in close proximity without aggression (personal observation). This is similar to patterns observed in other urban settings (e.g., Hedgehog Street 2025; Scott et al. 2023). Scott et al. (2023) reported that 51% of 143 hedgehog–fox encounters in urban gardens were neutral, with the remainder classified as agonistic. Within this agonistic group, no predation events were observed, although approximately 5% of interactions were considered potentially predatory. The remaining agonistic interactions were interpreted as competitive; hedgehogs and foxes frequently exploit similar food resources such as earthworms and other invertebrates. Injuries to adults, especially hind‐leg wounds, were consistent with attacks where foxes bite before hedgehogs curl into a defensive ball (Wildlife Online 2025). Comparable injuries were reported nearby on Hampstead Heath (Beasley et al. 2023). Other studies show mixed results: some suggest coexistence (Hitchcock et al. 2025), others avoidance (Gazzard et al. 2022). In arid Tunisia, foxes were confirmed as major hedgehog predators (El‐Farhati et al. 2022). Overall, the relationship between hedgehogs and foxes is context‐dependent, varying with habitat and food availability, and further behavioural research, especially on the motivation for foxes to attack hedgehogs, would help clarify the ecological dynamics of this relationship across parkland, urban and rural environments.

Road traffic deaths, commonly reported (Kristiansson 1990; Dowding et al. 2010; Haigh, O'Riordan, and Butler 2014; Moore et al. 2020; Wright et al. 2020), were rare in this study. Between 2015 and 2018, nine were recorded and none after 2018, likely reflecting lower population size and maybe reduced road crossings to and from the Zoo Car Park. Other causes of death included infection, trauma and drowning. Veterinary checks found no unusual disease or parasite loads. Exposure to second‐generation anticoagulant rodenticides (SGARs) from pest control near food outlets and the Zoo remains a possible sublethal factor (Gurnell et al. 2017; Dowding et al. 2010; Rasmussen et al. 2024; Miserez et al. 2025) but requires further study. Overall, recruitment was inconsistent and mortality high, leading to a progressive population decline that the current breeding rate could not offset.

### 4.3 Why Is the Population at a Tipping Point?

Two key factors appear to have driven hedgehog fluctuations in the Park: changes in distribution and predation pressure. Hedgehogs once occupied the entire Park but have not recolonised the southern and western areas since 2015. Despite management efforts, their continued absence suggests these areas are less suitable or that numbers are too low to support dispersal. Further study of habitat quality, prey availability and management practices would be valuable.

Human disturbance may also contribute. Although visitor data were not collected, observations suggest consistently heavy park use, peaking during COVID‐19 lockdowns when outdoor gatherings were permitted. Elsewhere, such disturbance has been linked to hedgehog stress (Berger et al. 2020). Regent's Park closes from dusk until 05:30 a.m., so human activity does not directly overlap with peak hedgehog activity. However, Beasley et al. (2023) found longer twilight activity in hedgehogs at sites with fewer people and dogs, indicating that disturbance effects at Regent's Park warrant further investigation.

Predation remains the most critical constraint on recovery. Foxes (and possibly dogs) kill juveniles and injure adults, often fatally, undermining recruitment and survival. Although road casualties and disease were relatively minor, these additional sources of mortality compound predation impacts. Given the population's small size, such pressures have disproportionate effects.

In summary, surveys over 10 years reveal a substantial decline in a closed urban hedgehog population, now at critically low levels. With numbers currently low, opportunities for detailed study are limited, but current work is focused on investigating the causes of decline and implementing measures to support population recovery.

## Author Contributions

**John Gurnell:** data curation (equal), formal analysis (lead), methodology (equal), writing – original draft (equal), writing – review and editing (equal). **Nigel Reeve:** conceptualization (lead), data curation (equal), methodology (equal), writing – review and editing (equal). **Bryony Cross:** project administration (lead), supervision (lead), writing – review and editing (equal).

## Funding

The project was established with the assistance of a private donation from the Meyer family.

## Conflicts of Interest

The authors declare no conflicts of interest.

## Data Availability

The hedgehog survey data that support this paper are available at Zenodo, https://doi.org/10.5281/zenodo.15812681 (Gurnell et al. 2025).

## Appendix A

**TABLE A1:** (a) Based on range‐centre zones from combined spring and autumn captures: (a) individuals either not recaptured (*S*) or recaptured (M) the following year, grouped by their initial capture zone. (b) long distance moves (> 244 m).

Zone	1		2		3		4		5		6		Zoo	
Years	*S*	*M*	*S*	*M*	*S*	*M*	*S*	*M*	*S*	*M*	*S*	*M*	*S*	*M*
**(a)**														
2014 to 2015	19	4	6	10	1	0	2	0	5	1	2	1	0	1
2015 to 2016	8	1	10	6	0	1	1	2	6	2	2	0	0	0
2016 to 2017	2	0	4	11	1	2	0	3	4	2	0	0	0	1
2017 to 2018	0	0	8	7	4	4	5	5	2	0	0	4	1	3
2018 to 2019	0	0	3	9	5	3	7	2	1	0	1	2	2	1
2019 to 2020	0	0	16	0	6	4	6	1	1	1	4	2	1	0
2020 to 2021	1	0	2	2	1	3	0	1	0	3	2	4	2	0
2021 to 2022	0	0	2	2	6	1	1	0	2	0	2	1	1	3
2022 to 2023	0	0	1	2	1	1	0	1	1	0	0	0	4	1

**(b)**				
Years	Sex	First zone	Second zone	Distance (m)
2014 to 2015	F	2a	1b	1139
2014 to 2015	M	7	5	1069
2014 to 2015	M	1a	1b	253
2015 to 2016	F	2a	2a	310
2016 to 2017	M	2b	0	842
2016 to 2017	M	5	4	657
2016 to 2017	M	2a	3	398
2016 to 2017	M	4	3	390
2016 to 2017	F	2a	2b	385
2016 to 2017	F	4	3	319
2017 to 2018	F	6	3	695
2017 to 2018	M	6	2a	577
2017 to 2018	F	4	6	507
2017 to 2018	M	4	2b	477
2017 to 2018	F	2a	4	459
2017 to 2018	F	2b	2a	431
2017 to 2018	F	2b	2a	392
2017 to 2018	F	4	2b	365
2017 to 2018	M	6	4	329
2017 to 2018	F	7	2a	279
2017 to 2018	M	3	4	273
2017 to 2018	F	6	6	263
2018 to 2019	F	3	5	636
2018 to 2019	M	4	3	310
2018 to 2019	M	6	6	265
2019 to 2020	M	6	2a	741
2020 to 2021	M	5	3	804
2021 to 2022	M	7	2a	483
2021 to 2022	M	2a	3	255
2022 to 2023	M	2a	3	303
